# Identification of definitive serum biomarkers associated with disease activity in primary Sjögren’s syndrome

**DOI:** 10.1186/s13075-016-1006-1

**Published:** 2016-05-14

**Authors:** Ayumi Nishikawa, Katsuya Suzuki, Yoshiaki Kassai, Yuumi Gotou, Maiko Takiguchi, Takahiro Miyazaki, Keiko Yoshimoto, Hidekata Yasuoka, Kunihiro Yamaoka, Rimpei Morita, Akihiko Yoshimura, Tsutomu Takeuchi

**Affiliations:** Division of Rheumatology, Department of Internal Medicine, Keio University School of Medicine, 35 Shinanomachi, Shinjuku-ku, Tokyo 160-8582 Japan; Inflammation Drug Discovery Unit, Pharmaceutical Research Division, Takeda Pharmaceutical Company Limited, 2-26-1 Muraokahigashi, Fujisawa city, Kanagawa 251-0012 Japan; Department of Microbiology and Immunology, Keio University School of Medicine, 35 Shinanomachi, Shinjuku-ku, Tokyo 160-8582 Japan

**Keywords:** Sjögren’s syndrome, Biomarker, Proteomics, Serum protein, Disease activity

## Abstract

**Background:**

In this study, we sought to identify definitive biomarkers associated with disease activity in primary Sjögren’s syndrome (pSS).

**Methods:**

Serum protein concentrations in pSS patients and healthy controls (HCs) were comprehensively screened using high-throughput proteomic analysis, and differentially expressed proteins were extracted. Correlation between differentially expressed proteins and European League Against Rheumatism Sjögren’s Syndrome Disease Activity Index (ESSDAI) scores was analyzed and disease activity-associated biomarkers were identified. These biomarkers were validated by enzyme-linked immunosorbent assay (ELISA) in a separate pSS cohort.

**Results:**

The serum concentrations of 1100 proteins were compared between 30 pSS patients and 30 HCs, with 82 differentially expressed proteins identified as pSS-associated proteins. Of these 82 proteins, 9 were identified as disease activity-associated biomarkers. These nine biomarkers underwent validation by ELISA in a separate pSS validation cohort (*n* = 58), with five proteins (CXCL13, TNF-R2, CD48, B-cell activating factor (BAFF), and PD-L2) subsequently being confirmed as candidate biomarkers. Of these five candidate biomarkers, CXCL13 exhibited the most significant correlation with the lymphadenopathy, glandular, and pulmonary domains of the ESSDAI. CXCL13, TNF-R2 and CD48 exhibited a positive correlation with the biological domain of the ESSDAI. TNF-R2 exhibited the most negative correlation with uptake in the submandibular gland on technetium 99m-pertechnetate salivary gland scintigraphy.

**Conclusions:**

Our approach successfully identified serum biomarkers associated with disease activity in pSS patients. These markers might be potential therapeutic targets in pSS patients.

**Electronic supplementary material:**

The online version of this article (doi:10.1186/s13075-016-1006-1) contains supplementary material, which is available to authorized users.

## Background

Primary Sjögren’s syndrome (pSS) is a systemic autoimmune disease characterized by dry eyes and dry mouth, and by systemic manifestations, such as general fatigue and fever, and damage to multiple organs [1]. Immunological abnormalities such as antinuclear antibodies (ANAs), antibodies to SS-A or SS-B, and hypergammaglobulinemia are often detected in pSS patients by laboratory tests [2, 3]. Infiltration of lymphocytes in salivary or lachrymal glands is typically observed in affected patients, which results in destruction and subsequent fibrotic changes [3–6]. However, the pathogenesis of pSS remains unclear due to the heterogeneity of clinical phenotypes and complex pathogenetic mechanisms. The identification of disease-associated molecular clusters or biomarkers will therefore help to clarify the complex pathogenesis of pSS.

Previous studies attempted to identify novel biomarkers that reflect pSS pathogenesis, using traditional proteomic approaches such as two-dimensional electrophoresis or mass spectrometry to characterize protein expression profiles in lachrymal or salivary fluid [7–14]. Most of these profiles consist of secretory proteins, enzymes, and highly abundant immune-related proteins such as albumin and β2-microglobulin (β2MG). However, given that the roles of these biomarkers in pathogenesis are unclear, they are not used at the clinical level.

B-cell-activating factor (BAFF), β2MG and myxovirus resistance protein A (MxA) were recently identified as biomarkers that correlate with European League Against Rheumatism (EULAR) Sjögren’s Syndrome Disease Activity Index (ESSDAI) scores [15–17], which is an objective method of evaluating clinical disease activity in clinical pSS research [1, 4, 18, 19]. BAFF belongs to the tumor necrosis factor family and levels are slightly higher in the serum of pSS patients with lymphoproliferative disorders or clonal B-cell expansion in the salivary glands than pSS patients without these disorders. Serum β2MG is significantly higher in patients with pSS with history of lymphoma than in the others. MxA is a key mediator of the interferon (IFN)-induced antiviral response and is tightly regulated by type I IFNs. MxA is associated with a systemic type I IFN signature in certain subsets of patients with pSS. These studies demonstrate the clinical significance of the three biomarkers associated with the ESSDAI.

Here, we extracted disease-related molecular clusters and definitive protein biomarkers associated with pSS disease activity as assessed by the ESSDAI score, utilizing a novel comprehensive high-throughput proteomics analysis of more than 1100 proteins. We also validated the candidate biomarkers by ELISA in a separate pSS validation cohort.

## Methods

### Patients and controls

A total of 88 patients with primary Sjögren’s syndrome (pSS) meeting at least one of the following criteria: the 2002 American-European criteria for SS (AECG) [20]; the 2012 American College of Rheumatology (ACR) classification criteria for pSS [21]; or the revised Japanese Ministry of Health criteria for the diagnosis of SS [22], who had provided written informed consent and were returning for follow up at Keio University Hospital, were enrolled from April 2011 to July 2014. Of these 88 patients, 30 were analyzed in the initial cohort and the remaining 58 in the validation cohort; 40 of 88 patients satisfied the AECG criteria, 61 satisfied the ACR criteria, and 54 satisfied the Japanese criteria.

Patients who were being treated with moderate to high doses of corticosteroids, immunosuppressants, or biological agents were excluded. Thirty healthy individuals who did not suffer from autoimmune diseases or were not receiving any drugs were included as controls. Information on patient demographics and clinical parameters were retrospectively collected from medical records. All procedures were approved by the medical ethics committee of Keio University Hospital and followed the tenets of the Declaration of Helsinki. All samples and information were collected after patients and controls gave written informed consent.

### Clinical and histological assessments

Disease activity in pSS was quantified based on the ESSDAI score. The ESSDAI score evaluates 12 domains. Each domain is divided into three to four levels according to the degree of activity and scored as 0 (no activity), 1 (low activity), 2 (moderate activity) or 3 (high activity) [23]. The following tests were used to objectively assess the dryness of the eyes: Schirmer’s test, Rose Bengal (RB) score test, and fluorescein clearance test. The gum test was used as an indicator of oral dryness, and technetium 99m-pertechnetate scintigraphy was used to assess salivary gland function using standard clinical methods. Histological analysis was conducted using hematoxylin-eosin staining of lip biopsy specimens.

### Serum isolation and storage

After blood samples were collected from donors in tubes with serum-separating agent, serum was immediately separated by centrifugation, and several aliquots were stored at −80 °C until use.

### Comprehensive high-throughput screening of serum protein concentrations

Serum protein concentration was measured using a Slow Off-rate Modified DNA Aptamer (SOMAmer)-based capture array (SOMAscan^TM^; SomaLogic, Inc., Boulder, CO, USA), which is a comprehensive high-throughput proteomics assay using an Agilent microarray readout that measures 1128 proteins [24, 25]. Briefly, 75 μl of serum from each sample was incubated with a mixture of the 1128 SOMAmer® reagents that specifically bind to each protein and was incubated in separate wells on a 96-well plate. Each protein SOMAmer complex was then biotinylated and captured by streptavidin beads. SOMAmer was then removed and measured in relative fluorescence units (RFU) based on the fluorescent SOMAmer hybridized to a complementary probe on custom microarray slides [26], and the level of RFU was then converted to serum protein concentration.

### Enzyme-linked immunosorbent assay (ELISA)

Disease activity-associated biomarkers positively that were correlated with the ESSDAI scores were applied to validation analysis in different cohorts using ELISA. Briefly, serum samples were separated and stored at −80 °C until analysis. After thawing, the assay was performed in accordance with the manufacturer’s instructions. Concentrations of those biomarkers were measured and quantified using a spectrophotometer (iMark Microplate Absorbance Reader, Bio Rad, CA, USA).

The following commercially available kits for ELISA were used: Human Ephrin Type-B Receptor 2 (EPHB2) ELISA Kit (CUSABIO, Wuhan, Hubei Province P.R., China), Quantikine® Human CXCL13/BLC/BCA-1 Immunoassay ELISA kit (R&D Systems, Minneapolis, MN, USA), Human CD48 ELISA Pair Set (Sino Biological Inc., Beijing P.R., China), β-2 Microgloblin Human SimpleStep ELISA^TM^ Kit, (Abcam, Tokyo, Japan), Soluble TNF Receptor II Human ELISA Kit (Abcam), RayBio® Human LAG3 ELISA Kit (RayBiotech, Inc., Norcross, GA, USA), DuoSet® ELISA Human CD163 and DuoSet® Ancillary Reagent Kit2 (R&D Systems), and ELISA Kit for Programmed Cell Death Protein 1 Ligand 2 (PDCD1LG2) (Cloud-Clone Corp., Houston, TX, USA). For detection of BAFF, human BAFF affinity-purified polyclonal antibody and human BAFF biotinylated affinity-purified polyclonal antibody (R&D Systems) were used as previously described [27].

### Algorithm for identifying disease-related molecular clusters and definitive serum protein biomarkers associated with disease activity

**Fig. 1:**
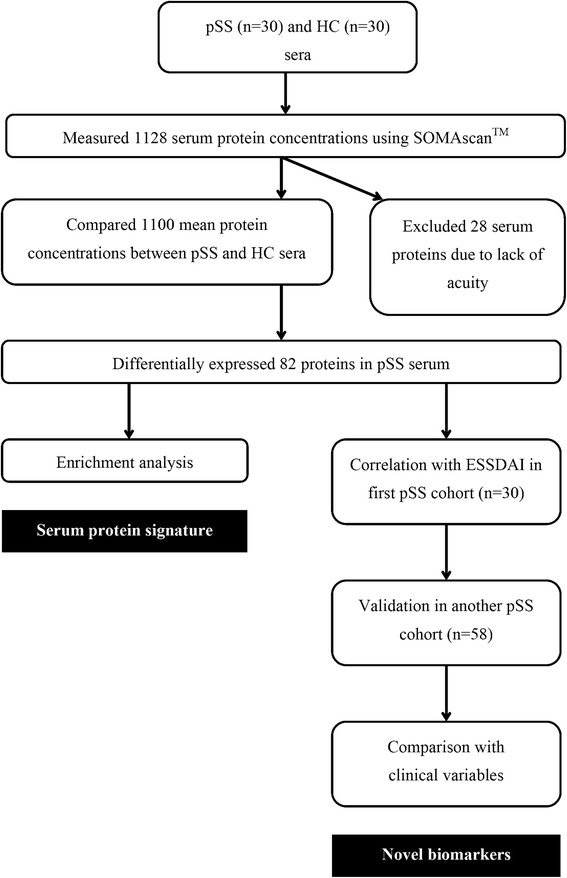
Overview of strategy for identification of serum proteins associated with disease activity and protein signature in patients with primary Sjögren’s syndrome (*pSS*). *HC* healthy controls, *ESSDAI* European League Against Rheumatism Sjögren’s Syndrome Disease Activity Index

Figure 1 shows the strategy for identifying disease-related molecular clusters and novel serum protein biomarkers associated with disease activity. A total of 1128 serum proteins in 30 pSS patients and 30 healthy controls (HCs) were comprehensively screened using SOMAscan^TM^. Twenty-eight serum proteins were excluded due to lack of acuity in measurement. Mean concentrations of the remaining 1100 proteins in the pSS and HC groups were compared. Differentially expressed serum proteins were selected as pSS-associated proteins based on the following criteria: *P* <0.05 for comparison of protein concentrations in patients with pSS and HCs using the Mann-Whitney *U* test, and fold-change in trimmed mean protein concentrations >1.2 or <0.83 in patients with pSS compared to HCs.

In enrichment analysis, biological interpretation of pSS-associated proteins was performed with original analysis software MoCo3 (Takeda Pharmaceutical Co., Ltd., Tokyo, Japan). First, the names of pSS-associated proteins were converted into gene symbols in public databases (further information available at http://www.ncbi.nlm.nih.gov/biosystems, http://genome.ucsc.edu/, http://www.ncbi.nlm.nih.gov/gap, http://www.genome.gov/gwastudies/, http://www.ncbi.nlm.nih.gov/, http://thebiogrid.org/, and http://hugenavigator.net/HuGENavigator/home.do) and a commercial database (BIOBASE Knowledge Library). MoCo3 evaluates the overlap between gene sets using Fisher’s exact test (a *P* value <0.01 is considered significant) and visualizes the statistical significant associations between them in a graph (network). The statistical methods and other computational algorithms of MoCo3 were as previously described [28].

For analysis of correlation between ESSDAI and serum protein concentration for continuous values, Spearman’s rho test (a *P* value <0.01 was considered significant) was used for analysis in the pSS initial cohort (*n* = 30) examined by SOMAscan^TM^, and Pearson’s correlation coefficient test (a *P* value <0.05 was considered significant) was used for the separate pSS validation cohort (*n* = 58) examined by ELISA. Finally, disease activity-associated biomarkers were compared with clinical variables, including clinical laboratory tests, clinical examinations, and imaging tests for salivary gland function using Pearson’s correlation coefficient test (a *P* value <0.05 was considered significant). All analyses were conducted using JMP® software, version 11.0 (SAS Institute Inc., Cary, NC, USA).

## Results

### Extraction of differentially expressed serum proteins in patients with pSS

Clinical characteristics of patients and controls are shown in Table 1. The ratio of women to men was almost equal in both patients with pSS and HCs. Mean age of patients with pSS was 61.1 ± 10.8 years, which was higher than that of HCs. Systemic activity in pSS was low overall, and the mean ESSDAI score was 2.6. Only one patient required treatment with corticosteroids during evaluation.

**Table 1:** Characteristics of patients and controls

Characteristic	Healthy controls (*n* = 30)	Patients with pSS (*n* = 30)
*Demographic*		
Female, n (%)	30 (100)	29 (97)
Age, mean (SD), years^*^	39.9 (9.4)	61.1 (10.8)
*Disease duration, mean (SD), years* ^*^	NA	4.7 (6.6)
*Clinical manifestation, n (%)*		
Ocular symptoms	NA	18 (60)
Oral symptoms	NA	21 (70)
Objectively assessed dryness	NA	24 (80)
Anti-SSA positivity	NA	24 (80)
Anti-SSB positivity	NA	14 (48)
Lymphocytic sialadenitis with focus score ≥1	NA	12 (40)
*ESSDAI, mean (SD)* ^*^	NA	2.6 (4.2)
*Treatment*		
Corticosteroid	NA	1 (3)
Immunosuppressant	NA	0 (0)

^*^Data are presented as mean ± standard deviation (SD) or number (%). *pSS* primary Sjögren’s syndrome, *ESSDAI* the European League Against Rheumatism Sjögren’s Syndrome Disease Activity Index, *NA* not applicable

In total 82 serum proteins that were differentially expressed in patients with pSS and HCs were extracted as pSS-associated proteins from 1128 proteins, based on a combination of statistical differences in serum concentration and fold-change in serum concentration. A total of 57 upregulated and 25 downregulated proteins were identified, along with the fold-change versus mean value of HCs, and the *P* value was calculated using the Mann-Whitney *U* test (Additional file 1: Table S1).

### Characteristics of disease-related molecular clusters in patients with pSS

To identify molecular clusters in the 82 pSS-associated proteins, enrichment analysis was applied as shown in Additional file 2: Figure S1. Characteristics of the serum protein signature in patients with pSS included the following molecular concepts: “extracellular region”, “chemokine signaling pathway”, “downstream of TNF-α”, “platelet activation”, and “platelet degranulation”. These molecular concepts were classified into immune response-related and platelet-related molecular clusters.

### Screening of proteins correlated with clinical disease activity in pSS

To extract disease activity-associated biomarkers, the correlation between serum protein levels and ESSDAI scores in patients with pSS was tested. Nine proteins were statistically extracted by Spearman’s rho test (Table 2) as follows: ephrin type-B receptor 2 (EPHB-2), C-X-C motif chemokine 13 (CXCL13), signaling lymphocytic activation molecule 2 (SLAMF-2, CD48), β2MG, BAFF, TNF receptor 2 (TNF-R2), lymphocyte activation gene-3 (LAG-3), cluster of differentiation 163 (CD163), and programmed cell death protein 1 ligand 2 (PD-L2).

**Table 2:** Serum proteins positively correlated with ESSDAI score in patients with pSS in first cohort (*n* = 30)

Protein	Spearman’s *ρ*	*P* value
EPHB-2	0.65	<0.0001
CXCL13	0.60	0.0005
CD48	0.56	0.0014
β2MG	0.55	0.0018
BAFF	0.52	0.0029
TNF-R2	0.51	0.0039
LAG-3	0.49	0.0058
CD163	0.49	0.0062
PD-L2	0.47	0.0081

Spearman’s rho correlation coefficient test was used to test correlation between 82 differentially expressed protein concentrations and the European League Against Rheumatism Sjögren’s Syndrome Disease Activity Index (*ESSDAI*) scores. *EPHB-2*, ephrin type-B receptor 2, *CXCL13*, C-X-C motif chemokine 13, *β2MG* β2-microglobulin, *BAFF* B-cell activating factor, *TNF-R2* TNF receptor 2, LAG-3, lymphocyte activation gene-3, *PD-L2* programmed cell death protein 1 ligand 2A *P* value <0.01 was considered significant

### Identification of novel disease activity-associated biomarkers positively correlated with ESSDAI scores in the pSS validation cohort

**Fig. 2:**
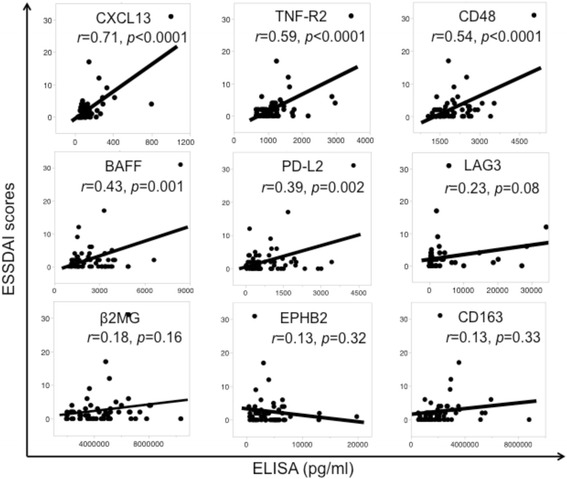
Correlation between European League Against Rheumatism Sjögren’s Syndrome Disease Activity Index (*ESSDAI*) scores and concentrations of nine screened serum proteins analyzed by ELISA in the validation cohort of patients with primary Sjögren’s syndrome (*n* = 58). The nine proteins were CXCL13, TNF receptor 2 (*TNF-R2*), CD48, B-cell activating factor (*BAFF*), programmed cell death protein 1 ligand 2 (*PD-L2*), lymphocyte activation gene-3 (*LAG-3*), β2-microglobulin (*β2MG*), ephrin type-B receptor 2 (*EPHB2*), and CD163. Pearson’s correlation test was used. The correlation coefficients (*r*) and *P* values are shown in the table. *P* <0.05 was considered significant

To confirm that the association between disease activity-associated biomarkers and ESSDAI scores was reproducible, a validation cohort consisting of serum samples from another 58 patients with pSS was analyzed. No marked differences in background characteristics were noted between the initial and validation cohorts (Table 3). Serum concentrations of nine candidates were measured using an ELISA and their correlation with the ESSDAI was statistically analyzed (Fig. 2). There was significant correlation between ESSDAI scores and CXCL13, TNF-R2, CD48, BAFF, and PD-L2 in both the initial and the validation cohorts of patients with pSS.

**Table 3:** Characteristics of patients in the validation and initial cohorts

Characteristic	Validation cohort (*n* = 58)	Initial cohort (*n* = 30)
*Demographic*		
Female, n (%)	57 (98)	29 (97)
Age, mean (SD), years^*^	60.3 (13.3)	61.1 (10.8)
Disease duration, mean (SD), years^*^	3.5 (5.8)	4.7 (6.6)
*Clinical manifestation, n (%)*		
Ocular symptoms	39 (67)	18 (60)
Oral symptoms	53 (91)	21 (70)
Objectively assessed dryness	48 (83)	24 (80)
Anti-SSA positivity	43 (74)	24 (80)
Anti-SSB positivity	24 (41)	14 (48)
Lymphocytic sialadenitis with focus score ≥1	10 (17)	12 (40)
ESSDAI, mean score (SD)^*^	2.6 (4.9)	2.6 (4.2)
*Treatment*		
Corticosteroid	1 (1.7)	1 (3)
Immunosuppressant	0 (0)	0 (0)

*Data are presented as mean ± SD or number (%). *Anti-SSA* anti-Sjögren’s syndrome antigen A antibodies, *Anti-SSB* anti-Sjögren’s syndrome antigen B antibodies, *ESSDAI* European League Against Rheumatism Sjögren’s Syndrome Disease Activity Index

### Association between disease activity-associated biomarkers and clinical characteristics of patients with pSS

To characterize serum biomarkers of clinical significance, the correlation between the five disease activity-associated biomarkers and various clinical parameters in the pSS validation cohort (*n* = 58) was assessed (Table 4). Notably, these five biomarkers were positively correlated with three domains of the ESSDAI, the lymphadenopathy, glandular, and pulmonary domains. In our pSS patient cohort, CXCL13 correlated significantly with the ESSDAI score and these three domains of the ESSDAI, and the strength of the correlation between the ESSDAI and TNF-R2, CD48, BAFF, and PD-L2, respectively, continued in this order. In addition, the serum concentrations of CXCL13, TNF-R2, and CD48 were positively correlated with that of immunoglobulin (Ig) G and the biological domain of the ESSDAI score.

**Table 4:** Clinical variables associated with five disease activity-associated biomarkers

Variable	N	CXCL13		TNF-R2		CD48		BAFF		PD-L2	
		*r*	*P*	*r*	*P*	*r*	*P*	*r*	*P*	*r*	*P*
*ESSDAI score*	58	0.7	<0.0001	0.59	<0.0001	0.54	<0.0001	0.43	0.001	0.39	0.002
Constitutional		NA	NA	NA	NA	NA	NA	NA	NA	NA	NA
Lymphadenopathy		0.69	<0.0001	0.54	<0.0001	0.58	<0.0001	0.58	<0.0001	0.5	<0.0001
Glandular		0.44	0.001	0.37	0.004	0.33	0.01	0.43	0.001	0.3	0.02
Articular		0.06	0.62	0.14	0.3	−0.03	0.8	0.11	0.39	−0.08	0.57
Cutaneous		NA	NA	NA	NA	NA	NA	NA	NA	NA	NA
Pulmonary		0.65	<0.0001	0.52	<0.0001	0.52	<0.0001	0.38	0.003	0.34	0.01
Renal		NA	NA	NA	NA	NA	NA	NA	NA	NA	NA
Muscular		NA	NA	NA	NA	NA	NA	NA	NA	NA	NA
Peripheral nervous system		NA	NA	NA	NA	NA	NA	NA	NA	NA	NA
Central nervous system		−0.001	0.1	0.01	0.94	−0.04	0.79	0.07	0.59	0.1	0.41
Hematological		0.17	0.2	0.1	0.47	0.02	0.9	−0.17	0.21	−0.13	0.32
Biological		0.5	<0.0001	0.42	0.001	0.45	0.0004	0.11	0.4	0.2	0.13
*IgG*	55	0.62	<0.0001	0.5	<0.0001	0.57	<0.0001	0.16	0.25	0.12	0.38
*C3*	52	−0.12	0.39	−0.06	0.69	−0.24	0.09	0.04	0.77	0.01	0.96
*C4*	52	0.13	0.37	0.24	0.08	0.18	0.2	0.2	0.15	0.13	0.37
*Schirmer test (mm/5 min)*	21	0.03	0.9	−0.28	0.23	−0.12	0.6	−0.38	0.09	0.23	0.32
*RB test (score)*	14	−0.04	0.9	0.18	0.55	0.17	0.57	0.05	0.86	−0.16	0.58
*Fluorescein (score)*	17	0.19	0.45	0.23	0.38	−0.12	0.65	0.3	0.23	0.2	0.44
*Gum test (ml)*	28	−0.18	0.35	−0.4	0.03	−0.28	0.15	−0.2	0.3	−0.02	0.91
*GS grade (score)*	23	0.08	0.72	0.13	0.54	0.23	0.29	0.28	0.2	0.23	0.3
*Scintigraphy*	40										
PG uptake ratio (%)		−0.18	0.26	−0.27	0.1	−0.01	0.95	0.01	0.94	−0.13	0.44
SMG uptake ratio (%)		−0.48	0.004	−0.52	0.001	−0.28	0.1	−0.43	0.01	−0.15	0.4
PG excretion ratio (%)		0.12	0.45	0.06	0.7	0.02	0.9	−0.05	0.77	−0.08	0.64
SMG excretion ratio (%)		0.05	0.79	0.26	0.18	0.09	0.62	−0.49	0.01	0.26	0.17

Candidate biomarkers and clinical variables are shown in the header row and left columns, respectively. Data are presented as Pearson’s correlation coefficients (*r*) and *P* values (*P*). A *P* value <0.05 was considered significant. *TNFR* TNF receptor, *BAFF* B-cell activating factor, *PD-L2* programmed cell death protein 1 ligand 2, *ESSDAI score* the European League Against Rheumatism Sjögren’s Syndrome Disease Activity Index (ESSDAI) score, *Schirmer’s test* Schirmer’s *I* test (mm/5 min), *RB test* Rose Bengal staining (score: van Bijsterveld score), *Fluorescein* fluorescein staining (score: van Bijsterveld score), *GS grade* Greenspan’s grade, *Scintigraphy* technetium 99m-pertechnetate salivary gland scintigraphy, *PG* parotid gland, *SMG* submandibular gland, *N* number of patients with primary Sjögren’s syndrome, *NA* not applicable

The associations were further investigated between these biomarkers, and clinical examinations, imaging tests for salivary gland function, and histological grade. TNF-R2 was negatively correlated with unstimulated salivary flow as assessed by the Gum test. CXCL13, TNF-R2, and BAFF were negatively correlated with uptake in the submandibular gland on technetium 99m-pertechnetate salivary gland scintigraphy, with TNF-R2 exhibiting the strongest correlation. In addition, only BAFF was negatively correlated with the excretion rate in the submandibular gland.

## Discussion

We conducted a comprehensive study of serum proteins in patients with pSS using the most recent and reliable high-throughput proteomics approach, with simultaneous screening of more than 1100 multiple proteins. We identified pSS-associated molecular clusters and validated disease activity-associated biomarkers in a larger cohort than in previous studies of biomarkers for pSS [7–15, 17]. We also analyzed the association between disease activity-associated biomarkers and clinical characteristics.

We first conducted enrichment analysis to clarify the presence of a distinct serum protein signature of pSS and found that the majority of pSS-associated proteins were involved in the immune response-related or the platelet-related molecular cluster. The immune response-related molecular cluster indicates altered immune responses, such as upregulated chemokine or cytokine expression and chemotaxis activation. This in turn suggests that an immune response is activated in the lesion of pSS, such as glandular and extra-glandular tissues. However, the platelet-related molecular cluster is associated with platelet activation, and its role in the pathophysiology of pSS remains unclear.

In this regard, Sarac et al. reported that patients with pSS with frequent episodic tension-type headache (FETH) had markedly decreased platelet serotonin levels (PSLs) and more common cerebral white matter signal hyperintensities (SHs) on brain magnetic resonance imaging than HCs. These findings appear to be associated with increased platelet serotonin release, indicating a more widespread cerebral vasculopathy in patients with pSS than in HCs [29, 30]. Tomlins et al. developed a molecular concept model of prostate cancer progression using similar enrichment analysis and further confirmed molecular concepts that correlated with known histological features of prostate cancer progression [28]. The pSS-associated molecular concepts obtained by our method might therefore be useful at a clinical level. Recently, Delaleu et al. reported ontology-term network mapping of salivary gland fluid proteins examined using Human Discovery Multi-analyte Profile 1.0 (Myriad RBM, Austin, TX, USA) and identified immune (mainly B-cell-related) responses, T cell chemotaxis, and macrophage activation pathways [31]. This profile was confirmed by analysis of molecules in saliva using a similar enrichment analysis, and this analysis showed the association with molecular clusters involved in formation of glandular pathophysiology. That both studies identified partially similar molecular clusters is of interest.

Various outcome measures used in previous clinical trials were based on glandular manifestations or symptoms, but not systemic manifestations. However, “activity indices” should contain both systemic and glandular features to evaluate the outcomes of new therapies. The ESSDAI was therefore developed as measure of disease activity in patients with systemic complications of pSS [18]. To date, ESSDAI is the only available disease activity index [32]. One of the strengths of our study includes the identification of biomarkers associated with the ESSDAI in patients with pSS, whose mean time of follow up was less than 5 years from diagnosis.

Our statistical extraction of surrogate biomarkers of the ESSDAI score also identified CXCL13, TNF-R2, CD48, BAFF, and PD-L2, which confirmed the findings of our validation study using a different cohort and methods. As these molecules are all involved in the immune response-related cluster, the immune response appears to be involved in this pathogenesis. These molecules might function as disease biomarkers for clinical follow up and as indicators of pSS pathogenesis. Very recently, CXCL13 was identified as a factor associated with the ESSDAI [33].

CXCL13 belongs to the CXC chemokine family. Follicular stromal cells, antigen-experienced T cells, and T helper (Th) follicular cells are all reported to produce CXCL13 [33–36], which recruits B cells to germinal centers. In patients with pSS, CXCL13 levels are upregulated in serum, saliva [37], and salivary gland tissue [38, 39]. Based on our results, CXCL13 is associated with the pathogenesis of pSS, such as immunoglobulin production, and is linked to the activity of lymphadenopathy, glandular manifestation, interstitial lung disease (ILD) and biological status of the salivary glands.

TNF-R2, also known as p75 and TNFRSF1B, is mainly expressed in certain lymphocyte populations, such as regulatory T cells and CD8^+^ T cells, endothelial cells, microglia, oligodendrocytes, cardiac myocytes, thymocytes, and human mesenchymal stem cells [40]. It is reported that TNF-R2 also presents in a soluble form (sTNF-R2) and that plasma sTNF-R2 levels are increased in patients with active systemic lupus erythematosus and Behçet’s disease [41–45]. In the examination of labial salivary gland tissues, Koski et al. [46] found that TNF-α, TNF-R1, and TNF-R2 were all expressed on vascular endothelial cells, ductal epithelial cells, and fibroblasts, but that only TNF-R1 was expressed on acinar end piece cells. TNF-R2 might therefore be associated with vascular or epithelial injury, which is a primary event in pSS.

CD48 is a member of the CD2 immunoglobulin superfamily, which includes SLAM proteins, and is expressed on the surface of lymphocytes and other immune cells, dendritic cells, and endothelial cells [47]. CD48 also exists in a soluble form (sCD48). Plasma sCD48 levels are elevated in patients with asthma, several infectious diseases including varicella, measles, and rubella, lymphoid leukemias, and arthritis [48–51]. However, the function of CD48 has not been clarified. Further investigation might reveal the association with the pathogenesis of pSS.

Several reports have been published on the role of BAFF in pSS. BAFF expression was increased in the salivary glands and the serum of patients with pSS [52]. Serum BAFF is particularly strongly upregulated in patients with pSS with lymphoproliferative disorders [15], and in patients with systemic lupus erthyematosus and rheumatoid arthritis [53–55]. Taken together with our present results, these previous findings suggest that BAFF might be associated with severe destruction of the salivary glands.

PD-L2 is a ligand of programmed cell death protein 1 (PD-1). It is reported that PD-L2 also has a soluble form [56]. Recent studies [56] have clarified significant roles of the PD-1/PD-L pathway in autoimmunity, including type 1 diabetes mellitus, systemic lupus erythematosus, rheumatoid arthritis and transplantation immunity, infectious immunity, and tumor immunity. PD-L2 might therefore modify PD-1/PD-L2 signaling and enhance immunoglobulin production, including autoantibodies, as PD-L2 expression has been observed on antigen-presenting cells (APCs) such as macrophages, dendritic cells, and activated T cells [57].

In addition, we extensively confirmed some characteristics of the molecules. To confirm whether these five proteins are specific for pSS, we compared serum concentration of them among four groups of patients (those with pSS and secondary Sjögren’s syndrome (sSS), sicca syndrome and HCs) as shown in Additional file 3: Figure S2. Increased serum levels of five proteins were observed in patients with pSS compared with HCs (*P* < 0.05). Only CD48 levels were increased in patients with pSS compared with sicca syndrome patients, and there was no difference in the serum levels of four proteins was found in patients with pSS and patients with sicca syndrome. Increased serum levels of TNF-R2 and PD-L2 were observed in patients with sicca syndrome compared with HCs (*P* < 0.05). We consider that these age-matched and sex-matched patients with sicca syndrome, who did not satisfy any criteria for SS, include the patients who are clinically suspected to have a high probability of having SS. That may be one of the reasons why there is no significant difference between pSS and other sicca syndromes. We also analyzed the association between the five proteins and age, but there was no strong correlation (Additional file 4: Figure S3).

Several limitations to the present study warrant attention. First, the size of our study cohort is a little small for identifying serum biomarkers in systemic autoimmune diseases, which are heterogeneous diseases. Second, our study cohort did not include patients with long-term follow up, hampering the confirmation of changes in levels of candidate biomarkers depending on activity.

## Conclusions

In conclusion, we comprehensively screened proteins related to disease activity and identified five clinically significant definitive serum biomarkers in patients with pSS. Further large-scale studies and analysis of the functional roles of these molecules are required to confirm their efficacy as markers for the evaluation of disease activity in pSS and the association with pathogenesis.

## Additional files

Additional file 1: Table S1. Differentially expressed serum proteins in patients with pSS compared to HCs. (DOC 85 kb) Additional file 2: Figure S1. Functional annotation of differentially expressed proteins in pSS patient sera. Nodes indicate molecular concepts or set of biologically related genes. Name of each node is indicated in *black text* on the node. The node size represents the proportion of differentially expressed gene symbols in the concepts (e.g., the “chemokine signaling pathway” and “extracellular region” concepts contain 14 and 58 genes, respectively). *Length of lines between nodes* represents degree of overlap between symbols. *Colored lines* indicate strength of functional relationship from strong to weak, as follows: *red*, *yellow*, *green* and *gray. Green nodes* indicate immune response-related molecular concepts, and *red nodes* indicate platelet-related molecular concepts. (TIF 9752 kb) Additional file 3: Figure S2. Serum levels of five proteins in pSS, sSS, sicca syndrome and HCs. The five proteins were CXCL13, TNF-R2, CD48, BAFF and PD-L2. Primary SS (*pSS*), *n* = 58; secondary SS (*sSS*), *n* = 6; other sicca syndrome, *n* = 13; healthy controls (*HCs*), *n* = 38. Differences in quantitative variables were analyzed by the Mann-Whitney *U* test when comparing two groups and by the Kruskal-Wallis test when comparing multiple groups. **P* value <0.05, which was considered significant. (TIF 33972 kb) Additional file 4: Figure S3. Correlation between levels of five serum proteins and age in the validation cohort of patients with pSS and HCs. A Patients with pSS, *n* = 58; B HCs, *n* = 30. Differences in quantitative variables were analyzed by the Pearson’s correlation coefficient test (*P* <0.05 was considered significant). The correlation coefficients (*r*) and *P* values (*p*) are shown. (TIF 33972 kb)

## Abbreviations

Anti-SSA: anti-Sjögren’s syndrome antigen A antibodies
anti-SSB: anti-Sjögren’s syndrome antigen B antibodies
β2MG: β2-microglobulin
BAFF: B-cell activating factor
CXCL13: C-X-C motif chemokine 13
ELISA: enzyme-linked immunosorbent assay
EPHB-2: ephrin type-B receptor 2
ESSDAI: European League Against Rheumatism Sjögren’s Syndrome Disease Activity Index
Fluorescein: fluorescein staining (score, van Bijsterveld score)
GS grade: Greenspan’s grade
IFN: interferon
Ig: immunoglobulin
LAG-3: lymphocyte activation gene-3
MxA: myxovirus resistance protein A
PD-L2: programmed cell death protein 1 ligand 2
PG: parotid gland
pSS: primary Sjögren’s syndrome. Schirmer’s test, Schirmer’s-*I* test (mm/5 min)
RB test: Rose Bengal staining (score, van Bijsterveld score)
Scintigraphy: technetium 99m-pertechnetate salivary gland scintigraphy
SLAMF2: CD48 signaling lymphocytic activation molecule 2
SMG: submandibular gland
TNF: tumor necrosis factor
TNF-R2: tumor necrosis factor receptor 2
