# Supplementary material for: Identification of definitive serum biomarkers associated with disease activity in primary Sjögren’s syndrome
Source: Arthritis Res Ther. 2016 May 14;18:106. doi: 10.1186/s13075-016-1006-1 (PMC4868006; doi:10.1186/s13075-016-1006-1)
Supplement: Additional file 1: Table S1. — Differentially expressed serum proteins in patients with pSS compared to HCs. (DOC 85 kb) [file 13075_2016_1006_MOESM1_ESM.doc]

**Additional file 1: Table S1. Differentially expressed serum proteins in pSS patients compared to HCs**

|  | **Fold change (vs. HCs)** | ***P*-value** |
| --- | --- | --- |
| **Up-regulated proteins** |  |  |
| *BAFF* | 9.40 | 0.0002 |
| *FSH* | 3.93 | 0.00001 |
| *CRP* | 3.14 | 0.0043 |
| *Lutenizing hormone* | 2.76 | 0.0001 |
| *HCG* | 2.66 | 0.0001 |
| *CD163* | 2.19 | 0.0002 |
| *LEAP-1* | 2.16 | 0.0034 |
| *I-TAC* | 1.82 | 0.00003 |
| *IP-10* | 1.72 | 0.00009 |
| *CXCL13* | 1.71 | 0.0033 |
| *LKHA4* | 1.62 | 0.00002 |
| *PARC* | 1.58 | 0.00002 |
| *vWF* | 1.57 | 0.0009 |
| *LAG-3* | 1.56 | 0.0001 |
| *TRAIL R4* | 1.53 | 0.0001 |
| *Apo B* | 1.53 | 0.0002 |
| *ENPP7* | 1.48 | 0.0023 |
| *MMP-12* | 1.47 | 0.0002 |
| *β2-microgloblin* | 1.47 | 0.00005 |
| *Chitotriosidase-1* | 1.47 | 0.0019 |
| *MCP-3* | 1.43 | 0.0023 |
| *IL-8* | 1.41 | 0.00001 |
| *SH21A* | 1.38 | 0.00007 |
| *TIMD3* | 1.36 | 0.0002 |
| *MMP-7* | 1.36 | 0.0054 |
| *Apo E* | 1.35 | 0.0476 |
| *DERM* | 1.33 | 0.0011 |
| *PBEF* | 1.33 | 0.0001 |
| *Elafin* | 1.32 | 0.0089 |
| *TPSB2* | 1.30 | 0.0004 |
| *Catalase* | 1.29 | 0.0006 |
| *RANTES* | 1.28 | 0.0062 |
| *TNFSF15* | 1.27 | 0.00003 |
| *TNF-R2* | 1.26 | 0.0003 |
| *Granzyme A* | 1.26 | 0.0125 |
| *CRK* | 1.25 | 0.0476 |
| *TFF3* | 1.25 | 0.0085 |
| *Macrophage mannnose receptor* | 1.25 | 0.0266 |
| *ILT-4* | 1.25 | 0.0019 |
| *Eotaxin* | 1.25 | 0.0001 |
| *hnRNP A2/B1* | 1.24 | 0.0228 |
| *IL-16* | 1.24 | 0.0022 |
| *Fractalkine/CXC3CL-1* | 1.23 | 0.0012 |
| *PHI* | 1.23 | 0.0003 |
| *Fibrinogen* | 1.22 | 0.0257 |
| *C5a* | 1.22 | 0.0110 |
| *iC3b* | 1.22 | 0.0001 |
| *ARI3A* | 1.22 | 0.0153 |
| *PD-L2* | 1.22 | 0.0160 |
| *CD48* | 1.21 | 0.0039 |
| *KYNU* | 1.21 | 0.0019 |
| *DC-SIGN* | 1.21 | 0.0153 |
| *EPHB2* | 1.20 | 0.0345 |
| *MP2K2* | 1.20 | 0.00004 |
| *Galectin-3* | 1.20 | 0.0054 |
| *CKAP2* | 1.20 | 0.0003 |
| *RS7* | 1.20 | 0.00001 |
| **Down-regulated proteins** |  |  |
| *IgD* | 0.21 | 0.0128 |
| *Angiotensinogen* | 0.48 | 0.0010 |
| *Carbonic anhydrase 6* | 0.54 | 0.00002 |
| *TIMP-2* | 0.56 | 0.0004 |
| *Cathepsin 5* | 0.72 | 0.0001 |
| *ADAM12* | 0.73 | 0.00001 |
| *EDAR* | 0.75 | 0.0060 |
| *CK-MB* | 0.75 | 0.0022 |
| *α2-Antiplasmin* | 0.75 | 0.00003 |
| *CYTT* | 0.76 | 0.0042 |
| *6-phosphogluconate*  *dehydrogenase* | 0.77 | 0.0068 |
| *Kallistatin* | 0.77 | 0.0050 |
| *PF-4* | 0.78 | 0.0008 |
| *BOC* | 0.79 | 0.0001 |
| *CYTN* | 0.79 | 0.0110 |
| *CTAP-3* | 0.79 | 0.00003 |
| *NAP-2* | 0.79 | 0.00001 |
| *CCL28* | 0.79 | 0.0476 |
| *ON* | 0.80 | 0.00002 |
| *Cystatin M* | 0.81 | 0.0007 |
| *PYY* | 0.81 | 0.0057 |
| *PDGF-AA* | 0.82 | 0.00003 |
| *LYNB* | 0.82 | 0.0173 |
| *PPID* | 0.82 | 0.0037 |
| *gp2b3a* | 0.83 | 0.0002 |
