# Supplementary figures and images for: Identification of definitive serum biomarkers associated with disease activity in primary Sjögren’s syndrome
Source: Arthritis Res Ther. 2016 May 14;18:106. doi: 10.1186/s13075-016-1006-1 (PMC4868006; doi:10.1186/s13075-016-1006-1)

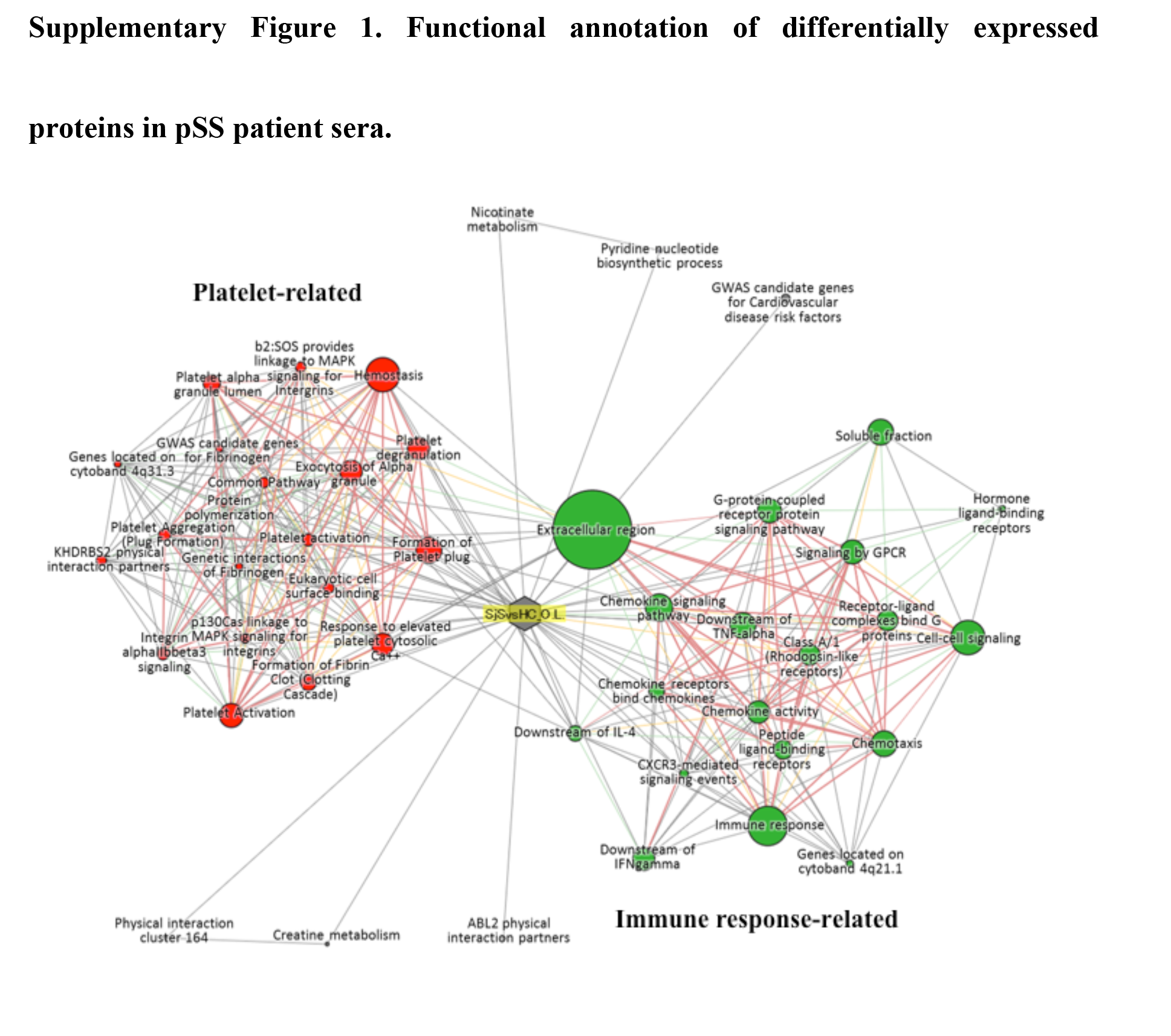

Supplement: Additional file 2: Figure S1. — Functional annotation of differentially expressed proteins in pSS patient sera. Nodes indicate molecular concepts or set of biologically related genes. Name of each node is indicated in black text on the node. The node size represents the proportion of differentially expressed gene symbols in the concepts (e.g., the “chemokine signaling pathway” and “extracellular region” concepts contain 14 and 58 genes, respectively). Length of lines between nodes represents degree of overlap between symbols. Colored lines indicate strength of functional relationship from strong to weak, as follows: red, yellow, green and gray. Green nodes indicate immune response-related molecular concepts, and red nodes indicate platelet-related molecular concepts. (TIF 9752 kb) [file 13075_2016_1006_MOESM2_ESM.tif]

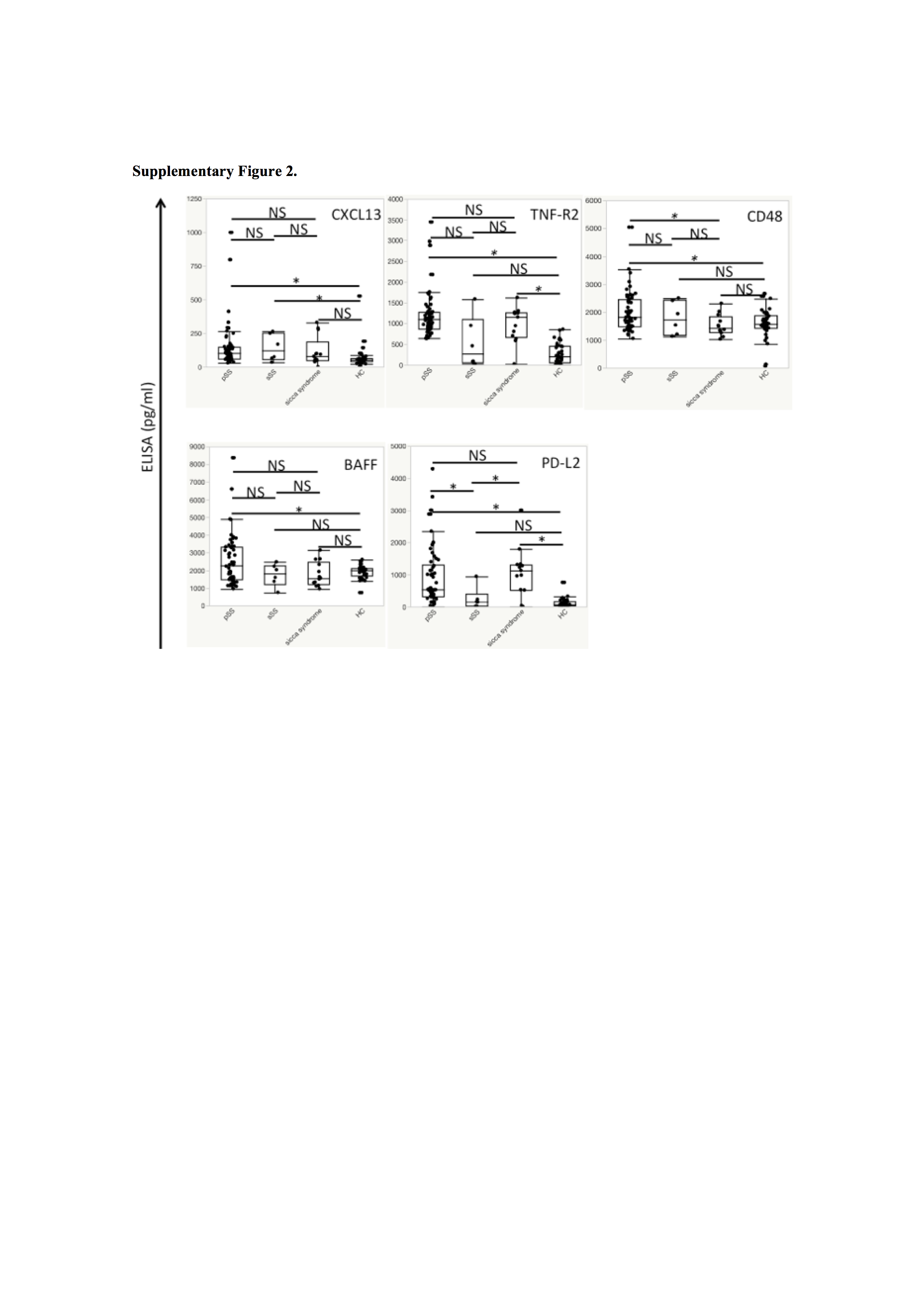

Supplement: Additional file 3: Figure S2. — Serum levels of five proteins in pSS, sSS, sicca syndrome and HCs. The five proteins were CXCL13, TNF-R2, CD48, BAFF and PD-L2. Primary SS (pSS), n = 58; secondary SS (sSS), n = 6; other sicca syndrome, n = 13; healthy controls (HCs), n = 38. Differences in quantitative variables were analyzed by the Mann-Whitney U test when comparing two groups and by the Kruskal-Wallis test when comparing multiple groups. *P value <0.05, which was considered significant. (TIF 33972 kb) [file 13075_2016_1006_MOESM3_ESM.tif]

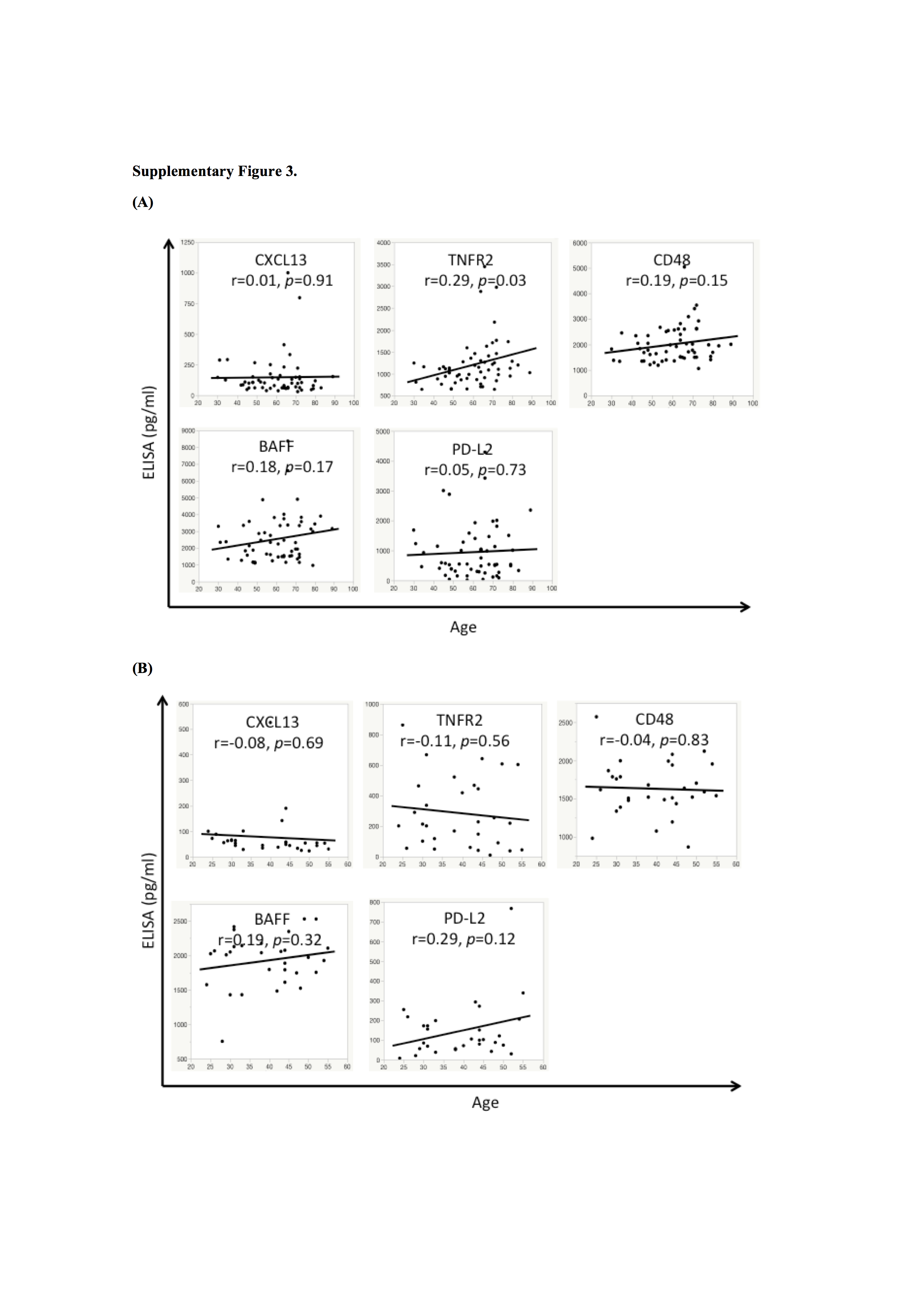

Supplement: Additional file 4: Figure S3. — Correlation between levels of five serum proteins and age in the validation cohort of patients with pSS and HCs. A Patients with pSS, n = 58; B HCs, n = 30. Differences in quantitative variables were analyzed by the Pearson’s correlation coefficient test (P <0.05 was considered significant). The correlation coefficients (r) and P values (p) are shown. (TIF 33972 kb) [file 13075_2016_1006_MOESM4_ESM.tif]
